# Lower Emotional Exhaustion among Employees Is Associated with Intentional Incorporation of Animals into Residential Care Settings

**DOI:** 10.3390/bs13050421

**Published:** 2023-05-16

**Authors:** Kimberly I. Tumlin, Elizabeth N. Riley, Olga Vsevolozhskaya, Michael Cull

**Affiliations:** 1 Department of Epidemiology and Environmental Health, College of Public Health, University of Kentucky, Lexington, KY 40506, USA; 2 Department of Health Management & Policy, College of Public Health, University of Kentucky, Lexington, KY 40506, USA; elizabeth.n.riley@uky.edu (E.N.R.); michael.cull@uky.edu (M.C.); 3 Department of Biostatistics, College of Public Health, University of Kentucky, Lexington, KY 40506, USA; vsevolozhskaya@uky.edu

**Keywords:** burnout, organizational culture, psychological safety, animals

## Abstract

Secondary effects of animal-integrated programming on residential care center (RCC) staff and organizational culture are not well understood. We explored emotional exhaustion among RCC employees both in facilities that incorporated animals and those that did not incorporate animals into the therapeutic environment. We conducted a survey throughout a large midwestern RCC system in the United States to determine relationships between organizational culture, emotional exhaustion, and the intentionality by which animals were incorporated into programming. Data were analyzed by examining associations between variables of interest using chi-square or *t*-tests, and linear mixed-effects modeling was used to identify potential confounding effects due to differences in children served within RCCs. Staff from RCCs that used animals intentionally reported lower emotional exhaustion (*p* = 0.006), and higher average workplace safety (*p* = 0.024) and psychological safety (*p* < 0.001). Integrating animals into RCC programming is associated with elements of a strong organizational culture. It is possible that animal-integrated programming has a positive impact on the facility culture and workforce, and/or that RCCs with strong pre-existing cultures are more likely to use animal-integrated programming.

## 1. Introduction

In May 2022, US Surgeon General Vivek Murthy issued an advisory warning of the high levels of burnout across a broad set of helping professions [1]. Beginning in January 2022, the World Health Organization (WHO) adopted the ICD-11 definition of burnout as a psychosocial syndrome that includes contexts of physical and emotional exhaustion, diminished accomplishment, and depersonalization (i.e., indifferent attitude towards work) from “chronic workplace stress that has not been successfully managed” [2]. Burnout was at high levels in the helping professionals prior to 2020, but the pressures created by the COVID-19 pandemic exacerbated the problem. This alert’s recommendations emphasize the need to focus on an organizational culture that values staff voices and psychological health; an organization’s culture is represented by its values, behaviors, and beliefs, and it lives in the daily routines and habits of its professionals.

Emotional exhaustion has been established as the core dimension of burnout [3]. Helping professionals may be at particularly high risk of emotional exhaustion, as it occurs when professionals feel they do not have the resources and emotional reserves needed to be effective [4]. The presence of emotional exhaustion, independent of depersonalization and diminished accomplishment, strongly predicts workforce outcomes such as attrition and engagement [5].

Residential care center (RCC) staff are part of public behavioral health, child welfare, and other helping systems. These facilities are often characterized by challenges with staff turnover rates as high as 25% [6]. Attrition in early years has been documented as a result of an inability to cope with workplace stressors and caseloads [7,8]. This early career turnover affects the transfer and preservation of organizational knowledge. Furthermore, social capital within the organization can be negatively impacted [7]. Such a loss is particularly critical in child welfare systems because of the need to provide sound and stable relationships with mentors to promote patient-centered care. Contributing to turnover, child welfare positions are often characterized by high caseloads and high job demands, both of which are compounded by the desire of staff to help each and every case. As such, these workers are considered high-risk for emotional exhaustion [9,10,11].

Despite recognition that emotional exhaustion is an important predictor of turnover [8,12,13], evidence-based practices that protect residential care center staff are a nascent field of inquiry. It is possible that a strong organizational culture can contribute to reduced turnover, particularly as it relates to protecting against emotional exhaustion [11,13,14]. Furthermore, depersonalization and withdrawal from social capital within an organization can influence the intention to leave [7].

The study of emotional exhaustion is complicated by multiple variables that ultimately influence a worker’s decision to leave their position. Some of these factors characterize the individual worker, including age, tenure in a position, and sex [8]. Other factors are inherent to a system, including an organization’s safety culture [15].

Organizational-level practices that improve hope [12], create trusting environments, contribute to commitment [7], and decrease levels of emotional exhaustion [13] shape the elements of a safety culture. Organizational culture can be enhanced by providing transparent working environments, models of supervision, training, and adequate resources for job demands, and by creating a culture in which staff can speak up about concerns [12,13,16]. Employment-based social capital and relationships are also thought to improve the employee ability to cope with traumatic experiences, reduce burnout syndrome, and thus improve retention rates [7]. In particular, adopting systemic approaches that contribute meaningful support and change are necessitated to ensure the prevention of emotional exhaustion [8,13,17].

Animal-assisted therapies are thought to improve relationships and aid in coping from traumatic experiences [10,18,19]. Animal interaction improves physiological responses primarily in the reduction of stress responses [20]. In this way, animals have been suggested as moderators in the creation of social interactions [21]. Specific to emotional exhaustion, the impact of therapy dog visits on nursing staff burnout as measured by the Maslach Burnout Index was previously evaluated [18]. These visits decreased feelings of stress and frustration, and improved coping from compassion fatigue and burnout [18]. Use of animals in the workplace to reduce stress has been suggested as a primary and secondary prevention strategy—meaning that stress is both prevented and reduced when animals are present [10]. Not only do animals promote human–animal attachment, but they also contribute to interpersonal relationships [18]. Abrahamson et al. (2016) [22] reported that social interactions with patients were improved following hospital staff interaction with dogs. In this way, the authors suggested that “flow on” effects should be studied further in staff, particularly when helping acute or critical care individuals.

Concurrent to addressing staff health and wellness, child welfare services are moving towards the adoption of clinical strategies that increase treatment effectiveness. Many have adopted the use of animals to aid in traumatic stress treatments of youth. Animal interaction has been associated with health for individuals, including physical, emotional, and psychological benefits [21,23,24,25,26,27,28]. Youth in residential services reported better relationships with schoolteachers and residential caregivers when receiving intentional animal-assisted therapy, although clinical symptoms of depression and anxiety remained [21]. Regarding workforce-related health outcomes, better relationships between youth and residential caregivers may indirectly affect worker emotional exhaustion when dogs and horses are used for youth trauma care [27]. Other work has demonstrated the reduction in blood cortisol among staff as a measure of physiological stress when dogs are part of the workplace environment [28]. However, neither of these associations define if and how the use of animals affects worker emotional exhaustion. Despite documented benefits of animals on human health [10,21,23], there is no research on how the secondary interaction and therapeutic intentionality of animal interaction may benefit RCC staff who are responsible for building and maintaining therapeutic environments.

To the best of our knowledge, no prior research has investigated factors in residential care centers that contribute to organizational culture and how animal incorporation affects worker perceptions. Given the paucity of data in this domain, we explored relationships between organizational culture data and the incorporation of animals into the workplace environment to (1) establish baseline organizational culture perceptions of workers to aid in contextualizing future studies on worker wellness programs, and (2) determine relationships between organizational culture constructs and the intentional incorporation of animals into the therapeutic milieu. Our hypothesis was that the intentional incorporation of animals into the therapeutic milieu will be positively associated with organizational culture constructs. Our sub-hypothesis was that child characteristics, caseloads, and worker tenure at the facility (e.g., histories of trauma, runaways) would dampen the positive associations of cultural constructs.

## 2. Materials and Methods

### 2.1. Data Source and Survey Design

Several data sources were used for the analyses. First, we conducted an annual anonymous quality improvement survey in a state-wide RCC licensing agency in the midwestern United States in 2021 (see Table S1). The survey was offered to RCC employees, including administrators and frontline staff, and it collected information on participant demographics, facility operations (e.g., types of programming), organizational culture, and emotional exhaustion. Vogus et al. (2016) utilized several scales to assess organizational culture in a child welfare population, including psychological safety [29] and mindful organizing [30]; these two scales were included in this quality improvement survey in the use of the six-item version of the psychological safety scale. We also included scales assessing individuals’ perceptions of physical safety in the workplace and on how connected participants felt to their coworkers [31]. Emotional exhaustion was assessed using four items from the Maslach Burnout Inventory [11,13]. The licensing agency contacted RCC administrators about participation in this quality improvement survey; facility administrators then distributed this survey to their staff for voluntary participation. Survey enrollment was open for one month in summer 2021.

Second, we collected two years of RCC-level administrative data from the survey timeframe (January 2019 through December 2020) to characterize facilities in terms of the number of children they serve, children’s age, gender, and race distributions, percent of recurrent running away and detention episodes among children they serve, and children’s levels of complex traumatic experience. Children’s complex trauma was measured using the Child and Adolescent Needs and Strengths (CANS) instrument [32]. RCC employees are required to be trained and certified to complete a CANS assessment, and the CANS must be completed within 30 days of a child’s RCC placement. The CANS was shown to have a very good internal consistency in measuring traumatic stress symptoms [33] based on the trauma-specific items and domains. The scoring system of the CANS is based on a four-point scale (0–3 ratings), and items are rated according to two primary criteria: the degree of strength or need and the degree of urgency for intervention (“action levels”) for immediate use in practice [34]. Actionable scores (ratings of 2 or 3) indicate a significant level of need, requiring a need for action in or a focus on treatment and service planning. For these data, we focused on the use of trauma-specific items as “ever” experienced for actionable scores (ratings of 2 or 3), and not on the actual CANS score. We used demographics of served children to describe RCC homogeneity.

### 2.2. Animal Use and Intentionality

The key predictor of interest was the use of animals in residential programming. Participants provided information on whether their facility used animals in residential programming, and potential responses included the following: “animals have no role in residential programming”; “animals are part of the environment, but not used or incorporated into care plans”; “animals are a part of care plans, when providers offer an opportunity to participate”; “animals are a formal part of the care plans through linkage to external programs”; or “animals are a formal part of the care plans on site.” Responses describing the use of animals in residential programming were first dichotomized into one of two categories: animals have no role in our residential programming, or any response that indicated animals were used in programming. We then further coded responses for how intentionally animals were integrated into the therapeutic milieu into two categories: animals used, but incidentally (combining “animals are part of the environment, but not used or incorporated into care plans” and “animals are a part of our care plans, when providers offer an opportunity to participate” responses), and animals used, formal and planned (combining “animals are a formal part of the care plans on site” and “animals are a formal part of the care plans through linkage to external programs” responses). These manipulations resulted in three categories of the use of animals: (1) RCCs with no animals; (2) RCCs with animals that are used incidentally; and (3) RCCs with animals that are used intentionally.

The key outcome variables were the organizational culture constructs determined using survey data (i.e., emotional exhaustion, mindful organizing, psychological safety, workplace safety, and workplace connectedness). Covariates included the RCC-level characteristics of the children they serve, including the percent of children that were ever “actionable” on a trauma-specific item while in care, the percent of children that ever ran away while in care, and the percent of children that were detained (e.g., youth held while awaiting court decisions, such as a disposition or placement) while in care.

### 2.3. Analytical Approach

We used descriptive statistics to estimate sample characteristics by RCC categories of animal use. We used the chi-square (or a *t*-test) to statistically examine the association between the key predictor and categorical (or continuous) variables of interest. We used a linear mixed-effects model (LMM) to perform an association test of animal use in a residential program vs. the RCC’s organizational culture constructs while accounting for possible confounding effects due to differences in the characteristics of the children served by these RCCs. Random effects in the LMM were used to account for the correlation among a respondent’s survey answers from the same RCC. A backward stepwise elimination procedure was employed to identify the most parsimonious LMM model. All *p*-values were two-sided. Analyses were performed using R statistical software, version 4.1.2 [35].

## 3. Results

### 3.1. Participant Characteristics

Characteristics of the quality improvement survey participants (*n* = 213 workers within 16 different RCCs located in a large midwestern state) are presented in Table 1. The majority of them (51%) were employees of RCCs that reported using animals intentionally in their programs. Survey participants who reported intentional animal use in their residential programming tended to have less variable working hours (~10% vs. ~31% working variable hours in RCCs with no animals, *p* < 0.001); worked for a shorter period of time at a facility (33% reported less than one-year tenure at the facility vs. 16% in RCCs with no animals, *p* = 0.006); were younger (19% 18–24 years old vs. 12% in RCCs with no animals, *p* = 0.036); were more likely to be White or Caucasian (90% vs. 55% in RCCs with no animals, *p* < 0.001); worked fewer hours per week (~41 h vs. ~44 h in RCCs with no animals, *p* < 0.001); had lower levels of emotional exhaustion (40.88 vs. 44.20 in RCCs with no animals, *p* < 0.001); and had higher levels of workplace safety (5.75 vs. 5.08 in RCCs with no animals, *p* < 0.001) and psychological safety (5.06 vs. 4.45 in RCCs with no animals, *p* = 0.005). Animal use in RCC programming also had a positive association with levels of workplace connectedness (5.31 in RCCs with intentional animal use vs. 5.03 in RCCs with no animals) and levels of mindful organizing (4.77 in RCCs with intentional animal use vs. 4.47 in RCCs with no animals), although these associations did not reach statistical significance (both *p* > 0.05). There were no statistically significant differences between job title, level of education, gender composition, and sexual orientation across different RCC staff.

### 3.2. Child Characteristics

Table 2 describes characteristics of children served by 16 different RCCs located in a large midwestern state in the two years (January 2019 through December 2020) prior to our study. RCCs with intentional animal use tended to serve younger children (27% 9–12 years old vs. 8% in RCCs with no animals, *p* < 0.001) who were less likely to be males (56% vs. 78% in RCCs with no animals, *p* < 0.001) and who were more likely to be White (69% vs. 52% in RCCs with no animal use, *p* < 0.001); they were less likely to have a runaway (13% vs. 31%, *p* < 0.001) or a detention (34% vs. 71%) episode, but more likely to have ever witnessed family violence (51% vs. 41%, *p* = 0.044) or experienced neglect (56% vs. 41%, *p* = 0.003). There were no statistically significant differences in the percent of children that experienced physical abuse (*p* = 0.701), sexual abuse (*p* = 0.343), emotional abuse (*p* = 0.203), or exploitation (*p* = 0.834) across different RCCs.

### 3.3. LMM Regression Analyses

In the LMM regression analyses, all models were adjusted for RCC employees’ characteristics (i.e., age, race, gender, the number of hours worked per week, and the work shift) and characteristics of children they served (i.e., child age at entry to services, percent males, percent White, the number of runaway and detention episodes, and the number of children experiencing trauma). A backward stepwise elimination procedure was then employed to identify the most parsimonious models. Table 3 summarizes final LMM regression results for the four main outcomes of interest. Employees of RCCs with animal use were less likely to experience emotional exhaustion (adjusted odds ratio (aOR) 0.20, 95% CI: 0.06–0.19, *p* = 0.003, in RCCs with incidental, and aOR = 0.38, 95% CI: 0.55–0.75, *p* = 0.006, in RCCs with intentional animal use relative to RCCs with no animals), and more likely to experience workplace safety (aOR = 1.95, 95% CI: 1.09–3.51, *p* = 0.024, in RCCs with intentional animal use relative to RCCs with no animals) and psychological safety (aOR = 3.11, 95% CI: 1.78–5.41, *p* < 0.001, in RCCs with intentional animal use relative to RCCs with no animals). The remaining two outcomes (“workplace connectedness” and “mindful organizing”) had no significant variability across different levels of animal use in the RCCs.

## 4. Discussion

Previous work has shown a connection between emotional exhaustion and psychological safety [17], and the positive secondary effects of animal interaction on staff [22]. Prior to this study, the effects of animal incorporation into the therapeutic milieu had not been explored among RCC staff. To the best of our knowledge, this is the first study to also report on how animals and the intentionality of animal incorporation is related to organizational culture constructs of psychological safety and emotional exhaustion. Intentional incorporation of animals was defined as the use of an animal (e.g., dog, cat, horse) with directed and intentional therapeutic value for the children served; effects on staff were secondary, or flow-on, as they were not the focus of the therapeutic intervention.

Results from this study revealed several key differences between RCCs that incorporated animals into their programming compared to those who did not. In this study, the participants were the staff members of the RCCs. To test our hypothesis, we first compared demographics (e.g., race, age, education) and work factors (e.g., position type, shifts, length of tenure, hours worked) among RCCs. We acknowledge general differences among RCCs. Staff from RCCs using animals intentionally had the highest proportion from first shifts. In addition, these staff worked the fewest hours. One-third of staff from RCCs with animals used intentionally were within their first year of tenure and were also the youngest group of individuals (i.e., less than 24 years of age). Staff from RCCs who did not use animals in any capacity had the highest proportion of staff over 45 years of age and represented the greatest proportion of Black/African Americans (43% versus 7% RCCs with animals, used incidentally, and 2% in RCCs with animals, used intentionally). It is possible that organizational cultures at RCCs that use animals intentionally have pre-existing strategies intended to retain young staff and reduce emotional exhaustion.

We next evaluated whether characteristics of children served in the RCCs, as the differences in child demographics, traumatic experiences of children, and prevalence of runaways, may impact emotional exhaustion and organizational culture [11,13,14]. Using the child demographics to omit variable bias, we completed an LMM regression analysis. In our sample, children entering RCCs who used animals intentionally represented the largest number of children served, were the youngest population, and represented the greatest proportion of children who experienced neglect and family violence. RCCs who did not use animals served children with the highest prevalence of runaways and those in detention. Staff from RCCs using animals reported lower scores on emotional exhaustion; a lower score indicates less exhaustion. We would expect this result given the differences in characteristics of children, as runaways and detention increase job demands [9].

Among those RCCs that reported incorporating animals intentionally into their programming, staff reported lower exhaustion scores, which was different from staff reports in RCCs without any animal incorporation. We also observed greater workplace safety and psychological safety in staff responses from RCCs with animals than from RCCs who do not use animals. These findings support our hypothesis that intentionality in animal incorporation will affect organizational culture constructs experienced by staff.

We then adjusted the regression model for employee work factors (e.g., hours worked and shift) and characteristics of children served (e.g., case load and ages). Staff from RCCs with animals still demonstrated lower emotional exhaustion, even when adjustments to the model were made, which is contradictory to our secondary hypothesis. Acknowledging that RCCs incorporating animals had younger staff with less tenure and worked fewer hours, we controlled for all staff- and child-level variables in one model. In the LMM analysis, the effects of intentional animal incorporation on organizational culture constructs remained, regardless of differences among staff age, tenure, shift worked, child ages, caseloads, or child runaways. These results suggest that we have captured effects associated with animal incorporation, and they are not effects from staff age, tenure, hours worked, or child characteristics.

In other care settings, the incorporation of animals also resulted in improvements in emotional exhaustion, previously reported in a small group of nursing staff (*n* = 24) at an internal medicine hospital unit [18]. Conversely, staff burnout and emotional exhaustion were not affected by nursing staff from an oncology unit [36], staff from a healthcare clinic within a VA hospital [37], nor nursing staff in a metropolitan hospital [22] when animals were part of an intervention program. None of these studies reported intentional therapeutic goals, nor an intentional approach to the type of interaction. For some, having animals present in the workplace might be considered a distraction or break from traditional work experiences, which can contribute to greater job satisfaction [10,38,39]. The nuanced use of animals and secondary effects on RCC staff require additional investigation into how and when animals are incorporated into the therapeutic milieu.

### 4.1. Theoretical Implications

Job demands, age, and tenure of work within the individual’s career affect turnover [6,7,8]. Dimensions of employment-based social capital contribute to child welfare worker attrition, particularly in early years [7]. Prior work with animal interventions and staff perceptions have suggested that the client perception of the organizational environment was also improved when animals were present [22]. Less experienced workers are exposed to traumas and clients (e.g., youth and adolescents) for which training cannot prepare. Coping mechanisms vary among these young workers and are often dependent on social capital at the organizational level, predominantly organizational commitment and supervisory support [7]. An important component of the incorporation of animal-assisted programming into an organization includes acceptance of the program and perceptions of staff on the benefits of buffering that animals provide when dealing with workplace stress [10]. Similarly, our results also suggest that organizations with younger staff and less workplace tenure may also be more accepting of comprehensive animal-assisted programming, and may be demonstrating greater organizational commitment and supervisory support.

There is good reason to believe that the culture in organizations and workforce experiences related to emotional exhaustion and physical and psychological safety are interrelated and influence professional practice. Previous work in settings serving child welfare youth found that the organizational culture was strongly linked to turnover and outcomes with families [15]. In a survey of child welfare professionals in Tennessee, Vogus et al. (2016) [13] found strong correlations between emotional exhaustion and psychological safety, and recent work at the National Partnership for Child Safety has established strong relationships between these constructs measured in the organizational assessments of child welfare teams. Professionals with higher levels of workplace connectedness are more psychologically safe, and psychologically safe professionals have higher retention rates, lower levels of emotional exhaustion, and better teamwork skills [40,41]. Finally, understanding the impact of organizational culture on constructs that predict turnover, such as emotional exhaustion, is crucial for improving practice. There are well-established connections between emotional exhaustion and psychological safety and turnover [42]. Workers in public child welfare services with less than 3 years of tenure are more likely to leave when poor communication and less organizational support exist [7].

In recent years, interventions with animals have gained attention as a means to promote healing from traumatic physical and psychosocial injury. However, there are distinct differences in how humans interact with companion animals versus other animals, and there are gaps in understanding if different approaches to the intervention are equally effective. Not all youth who may benefit from health services actually access them, so using animals may encourage engagement [43], particularly if research approaches preserve the integrity of the therapeutic environment. Primary reasons for choosing horse-assisted interventions by families and caregivers are for enhancing this emotional regulation and improving life functioning, particularly when secondary conditions exist [44]. When considering mounted activities, such as therapeutic riding or vaulting, horses provide increased opportunity for physical and emotional regulation over the use of dogs in interventions, and this inherent size difference merges concepts of both exposure treatment and affect-focused therapy. An environment in which staff and children interact with animals should be considered in conjunction with the intentionality of interaction in further research.

### 4.2. Practical Implications

When bringing in small animals for animal-assisted programming, such as dogs or cats, appropriate guidelines and procedures for animal visits are important to balancing risks and benefits. In a study evaluating an animal-assisted program in an acute care facility, where patients are at higher risk of infectious agents, staff reported reductions in stress, better social interaction, and perceptions of comfort when in the company of patients [22]. The authors highlighted the need for appropriate sanitation procedures to mitigate the risk of disease transfer. In a different study, which evaluated horse–human interaction environments across different microclimates [45], it was noted that 33% of facilities knew that volunteer workers had an allergy to horses. The environment in which humans interact with horses is different than standard behavioral treatments, which occur within an office or building structure. However, regardless of location, allergy to animals may pose additional risk, which should be considered in organizational protocols to ensure protections when considering animal-assisted interventions.

Within the current study, we asked what type of animal was part of the interaction, and these were primarily dogs within the RCC sites, and horses off-site. Responses for the type of animal represented in this study included hamsters, cats, dogs, horses, donkeys, and “other large” animals. We are not aware of any study that directly compares across types of animal-assisted interventions, and whether therapeutic intentionality was tested. A growing call for child welfare systems to learn from other safety-critical settings, such as healthcare, and for these systems to develop cultures of safety, is emerging [13,17,46,47]. This study responds to these calls by exploring the factors associated with an organizational safety culture in settings serving youth in child welfare and adds to the evidence of its benefits. Our findings demonstrated that the intentionality of animal use for client benefits resulted in a two-fold greater report of workplace safety and a three-fold greater report of psychological safety for the RCC staff. This result is compelling, as it suggests that secondary benefits of intentional animal-assisted programming are realized in RCC workers, regardless of variations in employee factors and characteristics of children served. This three-fold greater perception of psychological safety in staff from RCCs with intentional animal use suggests that organizations that utilize animals intentionally may inherently have more supportive organizational cultures. Relationships between the specific agency activities (i.e., incorporating animals into programming) and organizational culture constructs point to opportunities to reinforce habits supportive of a safety culture. Our findings connecting organizational culture with important workforce-related constructs that predict turnover (i.e., emotional exhaustion) and performance (i.e., psychological safety) point in the direction of specific team-based strategies focused on building supportive team structures. In chronically overworked and under-resourced systems, creating a culture that values safety and psychological well-being may be a key component to reducing turnover. There are likely many paths and strategies by which organizations can work to create and sustain a culture of safety, perhaps by engaging in innovative workplace practices, such as incorporating animals into programming.

### 4.3. Limitations

The results of this study should be contextualized within its important limitations. First, due to the cross-sectional nature of this study, we are not able to establish any kind of causal relationships between the incorporation of animals into RCC programming and the impact on the workforce or workplace culture to include animals in the therapeutic milieu. There are a number of possibilities that could help to explain these relationships. Perhaps the incorporation of animals into a workplace does have a direct impact on emotional exhaustion, workplace safety, and psychological safety scores for employees. It is also possible that organizations with pre-existing strong cultures and leadership are more likely to engage in innovative strategies, such as incorporating animals into programming. Future longitudinal work may help to elucidate the nature of these relationships, and it seems to be a promising direction for creating strategies to improve burnout syndrome in the residential care workforce. Second, because these data were drawn from an anonymous quality improvement project, we were not able to explore participation rates by agency, nor specific characteristics of agencies; it is therefore difficult to know how representative the data used in this study are as compared to the entire population of RCC staff for this system. Third, the majority of RCCs that did not use animals in their programming were from a large urban area in the state. While we attempted to control this using other covariates incorporated into the model, we cannot rule out the possibility that these effects were confounded by urbanicity. Finally, due to the nature of data collection using anonymous surveys, we were not able to clarify responses or glean more detailed information on the type and method by which animals were incorporated into RCC programming.

## 5. Conclusions

Emotional exhaustion was positively impacted by animal inclusion in the workplace environment in both intentional and non-intentional approaches. In addition, the intentional incorporation of animals into RCC programming provides secondary benefits of increased workplace safety and psychological safety for staff. Use of animals in the workplace provides an encouraging strategy for improving components of burnout syndrome in residential care workers.

## Figures and Tables

**Table 1:** Demographic information of RCC employees by RCC characteristic grouping.

	RCCs with No Animals *n* = 49 (23) No. (%)	RCCs with Animals, Used Incidentally *n* = 55 (26) No. (%)	RCCs with Animals, Used Intentionally *n* = 109 (51) No. (%)	*p*-Value
Job type				0.625
Direct service	36 (73.5)	38 (69.1)	83 (76.1)	
Administration	13 (26.5)	17 (30.9)	26 (23.9)	
Shift				<0.001
First shift	19 (39.6)	28 (50.9)	59 (56.2)	
Second shift	10 (20.8)	2 (3.6)	24 (22.9)	
Third shift	4 (8.3)	5 (9.1)	12 (11.4)	
Variable hours	15 (30.6)	19 (34.5)	10 (9.5)	
Tenure at facility				0.006
Less than 1 year	8 (16.3)	8 (14.8)	36 (33.0)	
1–5 years	27 (55.2)	18 (33.4)	40 (36.7)	
6–10 years	5 (10.2)	16 (29.1)	10 (11.0)	
11–20 years	6 (12.3)	6 (10.9)	15 (13.6)	
21+ years	3 (6.1)	6 (10.9)	6 (5.5)	
Age				0.036
18–24 years	6 (12.2)	2 (3.6)	21 (19.3)	
25–34 years	15 (30.6)	20 (36.4)	37 (33.9)	
35–44 years	9 (18.4)	18 (32.7)	30 (27.5)	
45–54 years	10 (20.4)	11 (20.0)	10 (9.2)	
55+ years	9 (18.4)	4 (7.3)	11 (10.1)	
Education				0.612
High school or GED	19 (39.6)	13 (24.5)	36 (33.0)	
Bachelor’s degree	19 (39.6)	25 (47.2)	47 (44.3)	
Master’s degree	10 (18.9)	14 (25.5)	20 (18.)	
Doctorate	0 (0)	1 (1.9)	3 (2.8)	
Race				<0.001
Black or African American	21 (43)	4 (7)	2 (2)	
White or Caucasian	27 (55)	45 (82)	98 (90)	
Other	1 (2)	6 (11)	9 (8)	
Sexual orientation				0.355
Heterosexual	32 (65)	42 (76)	82 (75)	
Non-Heterosexual	17 (35)	13 (24)	27 (25)	
Gender identity *				0.489
Female	25 (51.0)	39 (70.9)	67 (61.5)	
Male	22 (44.9)	14 (25.5)	31 (28.4)	
Average hours worked per week (hours)	44.20	44.58	40.88	<0.001
Average emotional exhaustion	4.19	4.01	3.31	<0.001
Average workplace safety	5.08	5.51	5.75	<0.001
Average workplace connectedness	5.03	5.18	5.31	0.325
Average mindful organizing	4.47	4.62	4.77	0.342
Average psychological safety	4.45	4.82	5.06	0.005

* A small percent of respondents declined to answer.

**Table 2:** Demographic information of children (*n* = 712) served within each RCC category for two years (2019–2020) prior to our study using actionable Child and Adolescent Needs and Strengths assessment values.

	RCCs with No Animals No. (%)	RCCs with Animals, Used Incidentally No. (%)	RCCs with Animals, Used Intentionally No. (%)	*p*-Value
Number of children served (% of sample)	*n* = 222 (31)	*n* = 206 (29)	*n* = 284 (40)	
Mean age at entry (95% CI)	14.7 (14.45–15.02)	14.1 (13.75–14.35)	13.4 (13.11–13.62)	<0.001
Age (years) group *				<0.001
9–12	17 (8)	44 (21)	77 (27)	
13–17	205 (92)	156 (76)	193 (68)	
Male	173 (78)	158 (77)	160 (56)	<0.001
Race				<0.001
White	115 (52)	145 (70)	195 (69)	
Black/African American	83 (37)	47 (23)	44 (15)	
Other/unknown	24 (11)	14 (7)	45 (16)	
Ever runaway (% of sample)	68 (31)	15 (7)	36 (13)	<0.001
Ever detention (% of sample)	158 (71)	71 (34)	97 (34)	<0.001
Ever physical abuse ^†^ (% of sample)	64 (29)	66 (32)	82 (29)	0.701
Ever sexual abuse ^†^ (% of sample)	39 (18)	37 (18)	63 (22)	0.343
Ever neglect ^†^ (% of sample)	91 (41)	109 (53)	158 (56)	0.003
Ever emotional abuse ^†^ (% of sample)	79 (36)	75 (36)	121 (43)	0.203
Ever witness to family violence ^†^ (% of sample)	91 (41)	89 (43)	146 (51)	0.044
Ever exploitation^†^ (% of sample)	23 (10)	22 (11)	26 (9)	0.834

* There were small proportions of younger (5–8 years, 2%) and older (18–20 years, <1%) children served by the RCCs. ^†^ The number of children that were ever actionable on a trauma-specific item while in care.

**Table 3:** Results of an association analyses between animal use and organizational culture constructs based on the LMM regression reported as adjusted odds ratios (95% CI).

	Emotional Exhaustion	Workplace Safety	Workplace Connectedness	Mindful Organizing	Psychological Safety
RCCs with no animals	Ref.	Ref.	Ref.	Ref.	Ref.
RCCs with animals, used incidentally	0.20 (0.06–0.19)	0.83 (0.45–1.61)	0.07 (0.03–2.07)	0.31 (0.77–2.08)	0.88 (0.49–1.58)
RCCs with animals, used intentionally	0.38 (0.55–0.75)	1.95 (1.09–3.51)	4.23 (0.96–5.27)	1.89 (0.97–2.33)	3.11 (1.78–5.41)

Note: All LMM models were adjusted for RCC employees’ characteristics (i.e., age, race, gender, the number of hours worked per week, and the work shift) and characteristics of children they served (i.e., age at entry, percent males, percent White, the number of runaway and detention episodes, and the number of children experiencing trauma).

## Data Availability

Data are available within the article. A confidentiality agreement (i.e., “Data Use Agreement”) restricts the public sharing of full data sets generated and/or analyzed during the current study.

## Supplementary Materials

The following supporting information can be downloaded at https://www.mdpi.com/article/10.3390/bs13050421/s1, Table S1: Animal-related and demographic question subset of annual organizational culture survey for residential care centers.
