# Supplementary material for: Lower Emotional Exhaustion among Employees Is Associated with Intentional Incorporation of Animals into Residential Care Settings
Source: Behav Sci (Basel). 2023 May 16;13(5):421. doi: 10.3390/bs13050421 (PMC10215697; doi:10.3390/bs13050421)
Supplement: Supplementary file 1 [file behavsci-13-00421-s001.zip › behavsci-2311285-supplementary.pdf]

Table S1. Animal-related and demographic question subset of annual organizational culture survey questions for residential care centers.

Animal Questions

Are animals incorporated into your residential programming?

- o Animals have no role in our residential programming
- o Animals are a formal part of the care plans on site
- o Animals are a formal part of the care plans through linkage to external programs
- o Animals are part of the environment, but not used or incorporated into care plans
- o Animals are part of our care plans when providers offer us an opportunity to participate

	This animal is not part of residential programming	This animal is housed at the residential facility	This animal is brought to the residential facility	Youth travel to the animal's location
**Are DOGS part of your residential programming?**				
**Are CATS part of your residential programming?**				
**Are FISH part of your residential programming?**				
**Are REPTILES part of your residential programming?**				
**Are SMALL FARM ANIMALS (e.g., sheep, chickens, ducks, goats) part of your residential programming?**				
**Are LARGE FARM ANIMALS (e.g., horses, beef or milk cattle) part of your residential programming?**				
**Are OTHER SMALL ANIMALS (e.g., hamsters, rabbits) part of your residential programming?**				
**Other (please specify)**				

Please select in which years animals have been a part of the residential programming (select all that apply):

- 2012
- 2013
- 2014
- 2015
- 2016
- 2017
- 2018
- 2019
- 2020
- 2021

What percentage of youth in your program participate with animals?

- 75-100%
- 50-74%
- 25-49%
- Less than 25%

How are youth in services participating in animal-assisted programs?

- Groups
- Individually

How often do youth interact with animals as a formal part of their care plan?

- Daily
- Weekly
- Monthly
- As needed (please specify)

Demographic Questions

Please select the job type that best describes you:

- Direct Service
- Administration

Which shift do you work?

- First Shift
- Second Shift
- Third Shift
- Variable hours (I do not work a specific shift)

How long have you worked at this facility?

- 0-3 months 4-6 months
- 7-9 months
- 10-12 months
- 1 to 3 years
- 4 to 5 years
- 6 to 10 years
- 11 to 15 years
- 16 to 20 years
- 21 years or more

How long have you worked in your current position?

- 0-3 months 4-6 months
- 7-9 months
- 10-12 months
- 1 to 3 years
- 4 to 5 years
- 6 to 10 years
- 11 to 15 years
- 16 to 20 years
- 21 years or more

Typically how many hours per week do you work?

40

(Place a mark on the scale above)

What is your age?

- 18-24
- 25-34
- 35-44
- 45-54
- 55+

What is your highest level of education?

- High school or GED
- Bachelors: BSW
- Bachelors: other field
- Masters: MSW
- Masters: other field
- Doctorate: DSW or PhD in social work
- Doctorate: other field

Please select the gender identify with which you most identify:

- Female
- Male
- Transgender Female
- Transgender Male
- Gender diverse, gender expansive, gender
- non-binary, or genderqueer
- Intersex
- None of these describe me
- Decline to answer

Please select the sexual orientation with which you most identify:

- Asexual
- Bisexual
- Gay or lesbian
- Heterosexual
- Pansexual
- Queer
- None of these describe me
- Decline to answer

Please select the race with which you most identify:

- American Indian or Alaska Native
- Asian
- Black or African American
- Native Hawaiian or Other Pacific Islander
- White or Caucasian
- Multiracial
- None of these apply to me
- Decline to answer

Organizational Culture Questions

Emotional Exhaustion

	Very Strongly Disagree	Strongly Disagree	Disagree	Neutral	Agree	Strongly Agree	Very Strongly Agree
I feel burned out from my work.	○	○	○	○	○	○	○
I dread getting up in the morning and having to face another day on the job.	○	○	○	○	○	○	○
I feel emotionally drained from my work.	○	○	○	○	○	○	○
I feel used up at the end of the work day.	○	○	○	○	○	○	○

Mindful Organizing—when completing this portion think about your "unit/team" as the facility in which you work

	Not At All	To A Very Limited Extent	To A Limited Extent	To A Moderate Extent	To A Considerabl e Extent	To A Great Extent	To A Very Great Extent
During a shift change, we usually discuss what to look out for	◯	◯	◯	◯	◯	◯	◯
When discussing specific situations or processes in our work, my team spends time identifying things we do not want to go wrong.	◯	◯	◯	◯	◯	◯	◯
My team discusses alternatives to improve how we go about our normal work activities.	◯	◯	◯	◯	◯	◯	◯
My team has a good understanding of each other's skills and talents.	◯	◯	◯	◯	◯	◯	◯
We discuss our unique skills with each other so we know who has relevant specialized skills and knowledge.	◯	◯	◯	◯	◯	◯	◯
My team talks about mistakes and ways to learn from them.	◯	◯	◯	◯	◯	◯	◯
When errors happen, my team discusses how we could have prevented them.	◯	◯	◯	◯	◯	◯	◯
When we attempt to solve a problem in my team, we take advantage of the unique skills of our colleagues.	◯	◯	◯	◯	◯	◯	◯
When a problem occurs in my unit/team, we all get together to figure out the solution.	◯	◯	◯	◯	◯	◯	◯
My team spends time identifying things we do not want to go wrong from a general and systemic perspective.	◯	◯	◯	◯	◯	◯	◯
During shift changes, important information is left out.	◯	◯	◯	◯	◯	◯	◯
DCF Child Welfare Licensing works with our team to discuss alternatives to improve how we go about our normal work activities.	◯	◯	◯	◯	◯	◯	◯

Psychological Safety—when completing this portion think about your "unit/team" as the facility in which you work

	Very Strongly Disagree	Strongly Disagree	Disagree	Neutral	Agree	Strongly Agree	Very Strongly Agree
If you make a mistake on my team, it is often held against you.	○	○	○	○	○	○	○
The people on my team value each other's unique skills and talents.	○	○	○	○	○	○	○
Members of my team are able to bring up concerns and challenges.	○	○	○	○	○	○	○
It is safe to take an interpersonal risk on my team.	○	○	○	○	○	○	○
On this team, people are sometimes rejected for having different ideas.	○	○	○	○	○	○	○
It is difficult to ask other members of this team for help.	○	○	○	○	○	○	○

Workplace Safety

	Very Strongly Disagree	Strongly Disagree	Disagree	Neutral	Agree	Strongly Agree	Very Strongly Agree
I feel safe going to and from my workplace on a daily basis (i.e., I feel safe in the community where the facility is located).	○	○	○	○	○	○	○
I feel safe in my work environment.	○	○	○	○	○	○	○
Employee safety is important in my organization.	○	○	○	○	○	○	○
I feel that I am protected in my work setting.	○	○	○	○	○	○	○

Workplace Connectedness

	Very Strongly Disagree	Strongly Disagree	Disagree	Neutral	Agree	Strongly Agree	Very Strongly Agree
I am able to work with my co-workers without sacrificing my principles.	◯	◯	◯	◯	◯	◯	◯
I use "we/us" rather than "they/them" when I talk about my co-workers to others.	◯	◯	◯	◯	◯	◯	◯
When I face challenges my co-workers provide the help and support I need.	◯	◯	◯	◯	◯	◯	◯
I have things in common with my co-workers.	◯	◯	◯	◯	◯	◯	◯
